# End-to-end automated body composition analyses with integrated quality control for opportunistic assessment of sarcopenia in CT

**DOI:** 10.1007/s00330-021-08313-x

**Published:** 2021-09-30

**Authors:** Sebastian Nowak, Maike Theis, Barbara D. Wichtmann, Anton Faron, Matthias F. Froelich, Fabian Tollens, Helena L. Geißler, Wolfgang Block, Julian A. Luetkens, Ulrike I. Attenberger, Alois M. Sprinkart

**Affiliations:** 1 Department of Diagnostic and Interventional Radiology, Quantitative Imaging Lab Bonn (QILaB), University Hospital Bonn, Venusberg-Campus 1, 53127 Bonn, Germany; 2 Department of Radiology and Nuclear Medicine, University Medical Centre Mannheim, Theodor-Kutzer-Ufer 1-3, 68167 Mannheim, Germany; 3 Department of Radiotherapy and Radiation Oncology, University Hospital Bonn, Venusberg-Campus 1, 53127 Bonn, Germany; 4 Department of Neuroradiology, University Hospital Bonn, Venusberg-Campus 1, 53127 Bonn, Germany

**Keywords:** Body composition, Tomography, X-ray computed, Deep learning, Quality control, Sarcopenia

## Abstract

**Objectives:**

To develop a pipeline for automated body composition analysis and skeletal muscle assessment with integrated quality control for large-scale application in opportunistic imaging.

**Methods:**

First, a convolutional neural network for extraction of a single slice at the L3/L4 lumbar level was developed on CT scans of 240 patients applying the nnU-Net framework. Second, a 2D competitive dense fully convolutional U-Net for segmentation of visceral and subcutaneous adipose tissue (VAT, SAT), skeletal muscle (SM), and subsequent determination of fatty muscle fraction (FMF) was developed on single CT slices of 1143 patients. For both steps, automated quality control was integrated by a logistic regression model classifying the presence of L3/L4 and a linear regression model predicting the segmentation quality in terms of Dice score. To evaluate the performance of the entire pipeline end-to-end, body composition metrics, and FMF were compared to manual analyses including 364 patients from two centers.

**Results:**

Excellent results were observed for slice extraction (*z*-deviation = 2.46 ± 6.20 mm) and segmentation (Dice score for SM = 0.95 ± 0.04, VAT = 0.98 ± 0.02, SAT = 0.97 ± 0.04) on the dual-center test set excluding cases with artifacts due to metallic implants. No data were excluded for end-to-end performance analyses. With a restrictive setting of the integrated segmentation quality control, 39 of 364 patients were excluded containing 8 cases with metallic implants. This setting ensured a high agreement between manual and fully automated analyses with mean relative area deviations of ΔSM = 3.3 ± 4.1%, ΔVAT = 3.0 ± 4.7%, ΔSAT = 2.7 ± 4.3%, and ΔFMF = 4.3 ± 4.4%.

**Conclusions:**

This study presents an end-to-end automated deep learning pipeline for large-scale opportunistic assessment of body composition metrics and sarcopenia biomarkers in clinical routine.

**Key Points:**

• Body composition metrics and skeletal muscle quality can be opportunistically determined from routine abdominal CT scans.

• A pipeline consisting of two convolutional neural networks allows an end-to-end automated analysis.

• Machine-learning-based quality control ensures high agreement between manual and automatic analysis.

**Supplementary Information:**

The online version contains supplementary material available at 10.1007/s00330-021-08313-x.

## Introduction

Body composition analyses aim to determine the quantity of connective tissue compartments. In addition to quantifying the amount of adipose and muscle tissue, recent work proposed methods to obtain additional information about a patient’s general condition by also determining the quality of skeletal muscle tissue in terms of fatty degeneration. Several studies demonstrated that these metrics determined from abdominal imaging provide prognostic implications in oncologic or cardiovascular diseases [1–8].

The amount of visceral and subcutaneous adipose tissue, as well as the amount and quality of muscle tissue, can be reliably determined from abdominal CT imaging. An opportunistic large-scale assessment in clinical routine has the potential to further enhance the understanding of the clinical value of body composition analyses in various diseases, e.g., for therapy decision and/or outcome prediction. Also, the establishment of gender-, age-, and ethnicity-specific norm values is only feasible through the widespread application of these analyses.

However, the determination of fat and muscle volume by manually annotating the region of interest by a radiologist is rather time-consuming, which currently prevents clinical routine application. Several studies have shown that area measurements of connective tissue compartments on a single slice at a certain lumbar level are highly correlated with total volume in the abdomen [9–11]. This led to greatly reduced annotation times for manual body composition analysis when applying a 2D— instead of a 3D approach. In recent years, several methods have been proposed for automating the required tissue segmentation step. It was a logical consequence that with the dominant rise of deep learning for image segmentation the previously manually segmented images were used to develop methods for automated segmentation by supervised learning [12–14]. However, manual interaction was still required for extraction of the single slice on which the automatic segmentation is performed. Only very recent work also includes deep-learning-based automated slice extraction as the next step for truly automated body composition analyses [15–17].

Moreover, to the best of our knowledge, there is currently no work that presents integrated quality control for both slice extraction and tissue segmentation. This still leaves one factor that represents an additional human effort in opportunistic analysis, namely identifying cases where the algorithm fails. Automatic determination of the predictive uncertainties can help identify cases with low-quality analyses and can additionally be used to monitor the performance of an autonomous system during deployment, as suggested for machine learning operations to manage deep learning life cycles. This can also help to detect changes in the data and to raise a warning in case of domain shifts.

Hence, the aim of this study was to develop an automated body composition analysis for abdominal CT with integrated quality checks and to evaluate the end-to-end performance of the proposed pipeline on dual-center test data.

## Material and methods

### Overview

**Fig. 1:**
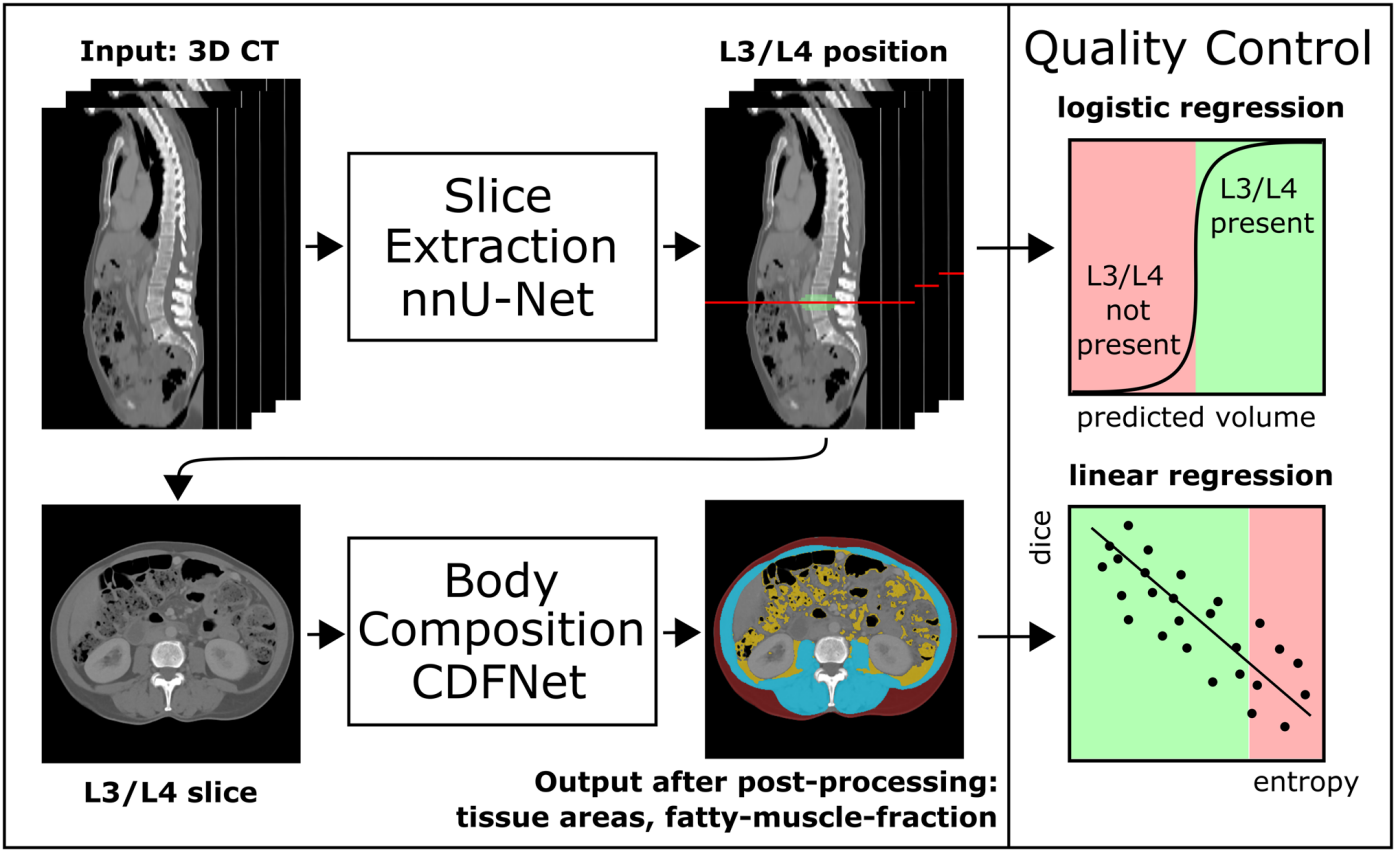
Schematic representation of the presented pipeline for autonomous body composition analysis. Input of the pipeline is a 3D CT scan. In the first part, a 3D convolutional neural network (CNN) was employed for slice extraction using nnU-Net. In the second part, a competitive dense fully connected CNN (CDFNet) is applied for segmentation of the body compartments. Classical machine learning methods were employed for integration of quality control steps. For the slice extraction part, a logistic regression model was developed that classifies the presence of L3/L4 lumbar level in the 3D CT scan. For segmentation of the different tissues, a linear regression model was established that predicts segmentation quality in terms of the Dice score

Figure 1 shows an overview of the developed pipeline. In the first part, a single slice at the L3/L4 lumbar level is extracted from a 3D CT scan. In the second part, the extracted 2D image is segmented into three compartment classes: visceral and subcutaneous adipose tissue (VAT, SAT) and skeletal muscle (SM). The fatty muscle fraction (FMF), a quantitative marker for fatty muscle degeneration, is determined in a subsequent post-processing step [1, 6]. For both deep-learning-based slice extraction and segmentation, classical machine learning methods were employed for integration of quality control steps that capture the predictive uncertainty during deployment.

**Fig. 2:**
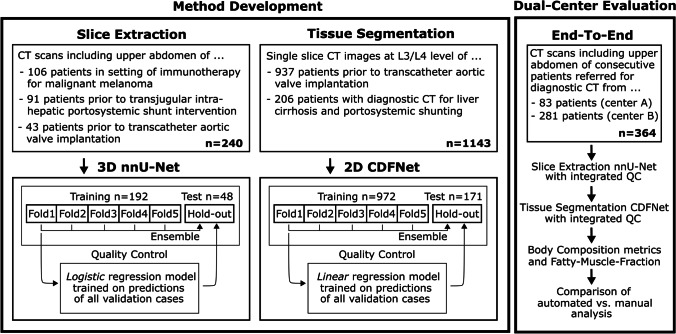
Overview of the data sets used for method development and evaluation. The nnU-Net employed for extraction of a single slice at L3/L4 level from a 3D CT scan and the CDFNet for tissue segmentation of the 2D CT slices were developed on two different datasets. Both methods were fivefold cross-validated and an ensemble of the cross-validated models was tested on the hold-out data. The regression models for integrated quality control (QC) were developed on the validation data of the cross-validated models and were also tested on the hold-out data. Finally, the entire pipeline of slice extraction, tissue segmentation, and quality control was evaluated end-to-end on the dual-center test data and compared against manual analyses

Slice extraction and tissue segmentation were developed independently. To evaluate the end-to-end performance of the entire pipeline, automatically extracted body composition metrics and FMF were compared with manual analyses on an unselected dual-center test set. Figure 2 provides an overview of the data sets used for method development and evaluation.

### Method development for slice extraction

#### Dataset

With institutional review board approval, written informed patient consent was waived because of the retrospective nature of all parts of the study. Retrospectively derived 3D CT scans of 240 patients (94 female, mean age 65 ± 14 years) referred for diagnostic CT including imaging of the upper abdomen acquired at eight different CT scanners were used for development of the slice extraction method. Of these patients, 43 received CT before undergoing transcatheter aortic valve implantation, 91 before transjugular intrahepatic portosystemic shunt intervention, and 106 patients received CT in the setting of immunotherapy for malignant melanoma.

The ground truth was generated by a board-certified radiologist (A.F.) by manually defining the center of the L3/L4 vertebral disk with an in-house tool (Matlab, Mathworks). Data were randomly split into a training set (*n* = 192, 80%) and a hold-out test (*n* = 48, 20%) set. The method was additionally tested on dual-center test data (described below).

#### Model

The extraction of a single slice at L3/L4 lumbar level was formulated as a segmentation task. A 3D U-Net architecture was trained using the nnU-Net framework, which has achieved high-performance values for various medical segmentation tasks and has the advantage of automatically adapting to different input sizes [18]. This is a relevant feature for the slice extraction task since the input are CT scans with a wide variety of scan lengths. The label map for training of the network was generated by applying a Gaussian distribution to the coordinates of the L3/L4 vertebral disk and binarizing the resulting probability map by a threshold [19]. Further details on image pre-processing, augmentation, and experimental design can be found in Supplement S1. For training, fivefold cross-validation was used and testing was performed with an ensemble of the cross-validated models.

#### Quality control

After training of the slice extraction method, a logistic regression model was built to automatically identify 3D CT scans that do not include the L3/L4 lumbar level. To obtain a balanced distribution of images with and without the L3/L4 lumbar level, for each 3D CT scan of the training, hold-out and dual-center test set, a cropped version was created. The logistic regression model was trained based on the predicted volume of all validation cases of the cross-validated slice extraction nnU-Net and applied to all test sets. Additional information about cropping and feature selection can be found in Supplement S2.

### Method development for tissue segmentation

#### Dataset

For the development of the tissue segmentation method (VAT, SAT, SM), retrospectively derived single slice images at the L3/L4 lumbar level from 1143 patients (559 female, mean age 77 ± 11 years) were used. 937 patients underwent pre-interventional CT for transcatheter aortic valve implantation and 206 patients underwent diagnostic CT for liver cirrhosis with portosystemic shunting. The dataset intentionally included a high number of patients with anasarca (19.2%), ascites (9.4%), or both anasarca and ascites (6.5%). The ground truth of the segmentation was defined by manual drawing and was also used to train a different CNN in a previous work, where additional details on the dataset are reported [13].

The data for method development were randomly split into a training set (*n* = 972, 85%) and hold-out test (*n* = 171, 15%) set. The method was additionally tested on dual-center test data (described below).

#### Model

A 2D competitive dense fully convolutional network (CDFNet), which has shown promising results for body composition analysis in magnetic resonance imaging, was used for tissue segmentation [20]. This architecture is proposed as an extension of the Dense-UNet architecture by max-out activation units. In a CDFNet, feature maps are generated by element-wise selection of the maximum values of previous feature maps, which has been shown to have a positive effect on performance and generalizability compared to unselective concatenation [20–22]. Further details on image pre-processing, augmentation, experimental design and computation of the fatty muscle fraction are provided in Supplement S3.

For training, fivefold cross-validation was used and testing was performed with an ensemble of the cross-validated models.

#### Quality control

To assess the predictive uncertainty of the segmentation during employment, a linear regression model was developed that predicts the segmentation Dice score for the muscle class based on the average entropies of the probability maps. This metric is proposed by a recent work as a feature to estimate quality of medical image segmentation and to detect out-of-distribution samples and ambiguous cases [23]. Although this method could be applied to all tissue classes, we focused on the muscle class because we consider it the most important class for the assessment of sarcopenia.

The linear regression model was trained with the predictions of all validation cases of the cross-validated tissue segmentation CDFNet and tested on all test sets.

### Dual-center test data and end-to-end evaluation

The entire pipeline was finally evaluated end-to-end, i.e., from 3D CT scan to extracted body composition metrics. The automatically determined tissue areas and the fatty muscle fraction were compared with the manually determined values. For this purpose, 3D CT scans of consecutive patients referred for diagnostic CT including imaging of the upper abdomen were retrospectively retrieved from two centers.

- Center A: 83 (41 females, mean age 60 ± 15 years) patients were used as internal test data from the Department of Diagnostic and Interventional Radiology, University Hospital Bonn. Data were acquired at four different CT scanners.
- Center B: 281 (111 females, mean age 63 ± 16 years) patients were used as external test data from the Department of Radiology and Nuclear Medicine, University Medical Centre Mannheim. Data were acquired at three different CT scanners.

In this data set, 10 patients had metallic implants. However, in the end-to-end evaluation, these cases were intentionally not excluded. For demonstration of the tissue segmentation quality control, a restrictive setting was applied excluding 10% of the cases with lowest predicted Dice score of the muscle class. End-to-end performance is reported for both included and excluded cases.

The ground truth for slice extraction and tissue segmentation was labeled by a radiology resident (B.W.) and a board-certified radiologist (A.F.). All labels of the radiology resident were validated by the board-certified radiologist.

Additional information on dual-center test data can be found in Supplement S5.

## Results

**Fig. 3:**
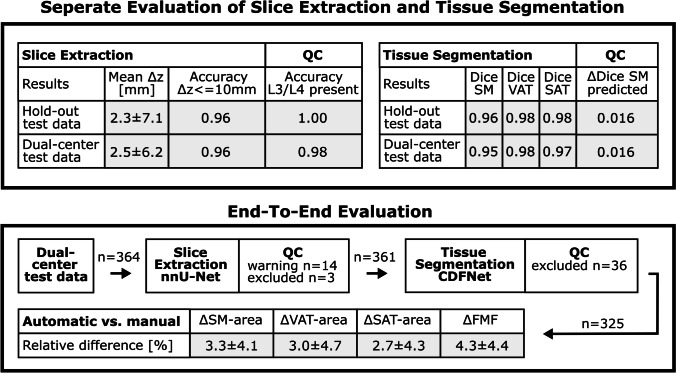
Summary of results: separate analyses of slice extraction, tissue segmentation, and respective quality control (QC), as well as agreement between end-to-end automated and manual area measurements of skeletal muscle (SM), visceral adipose tissue (VAT), subcutaneous adipose tissue (SAT), and the fatty muscle fraction (FMF)

A summary of the results can be found in Fig. 3.

### Slice extraction

The mean deviations between the predictions of the ensemble of cross-validated slice extraction models and the manually defined ground truth were Δ*z* = 2.27 ± 7.08 mm for the hold-out test data and Δ*z* = 2.46 ± 6.20 mm for the dual-center test data. Considering an acceptable deviation of up to 10 mm, 96% of the extracted slices of the hold-out test set and 96% of the dual-center test data were extracted at the correct level. The mean deviations are listed separately for all test sets in Table 1.

**Table 1:** Mean *z*-deviation (Δ*z*) and slice extraction accuracy for different tolerance margins obtained with the cross-validated nnU-Net ensemble for the hold-out test set and for the additional test data from center A and center B

Slice extraction	Mean, Δ*z* [mm]	Accuracy, Δ*z* = 0 mm	Accuracy, Δ*z* < = 5 mm	Accuracy, Δ*z* < = 10 mm
Hold-out	2.27 ± 7.08	0.79	0.96	0.96
Center A	3.35 ± 4.10	0.51	0.88	0.99
Center B	2.19 ± 6.70	0.85	0.96	0.96

### Tissue segmentation

The ensemble of fivefold cross-validated CDFNet models achieved excellent Dice scores, both on the hold-out test data (SM: 0.96 ± 0.02, VAT: 0.98 ± 0.02, SAT: 0.98 ± 0.01) and on the dual-center test data (SM: 0.95 ± 0.04, VAT: 0.98 ± 0.02, SAT: 0.97 ± 0.04). Table 2 lists the Dice scores separately for each test set.

**Table 2:** Dice scores for segmentation of skeletal muscle (SM), visceral adipose tissue (VAT), and subcutaneous adipose tissue (SAT) obtained with the cross-validated CDFNet ensemble for the hold-out test set and for the additional test data from center A and center B

Tissue segmentation	Dice score, SM	Dice score, VAT	Dice score, SAT
Hold-out	0.958 ± 0.023	0.981 ± 0.015	0.982 ± 0.012
Center A	0.959 ± 0.021	0.981 ± 0.012	0.979 ± 0.038
Center B	0.944 ± 0.039	0.974 ± 0.027	0.969 ± 0.037

### Quality control

**Fig. 4:**
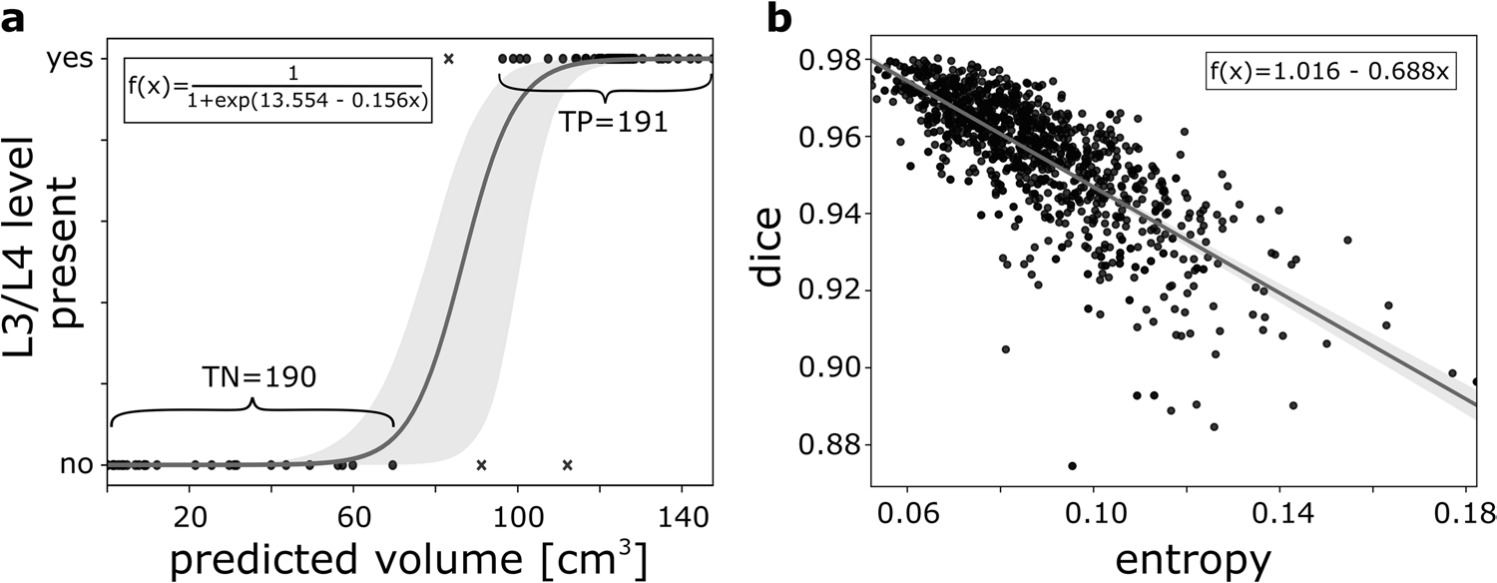
Models trained for quality control: **a** Based on the predicted volume of the nnU-Net employed for slice extraction, a logistic regression model was trained to predict the presence of the slice at L3/L4 lumbar level in the 3D CT scan. **b** For prediction of the tissue segmentation quality in terms of the Dice score, a linear regression model was trained based on the entropy of the probability map of the CDFNet for the muscle class. Both regression models were built on features derived from cross-validation data of slice extraction and tissue segmentation, respectively. Gray areas represent the 95% confidence intervals

Figure 4a shows the logistic regression model developed for identifying 3D CT scans that do not contain the L3/L4 level. High accuracy was observed for predicting the presence of the L3/L4 level in the original and cropped versions of the hold-out test data (100%) and also on the dual-center test data (center A: 99%, center B: 98%). Sensitivity and specificity were 97% and 99% for the dual-center test data.

The linear regression model developed for integrated quality control of the tissue segmentation is shown in Fig. 4b. Mean differences between the observed and the predicted Dice score for the hold-out test data were 0.016 ± 0.016 (SM), 0.005 ± 0.005 (VAT), and 0.008 ± 0.010 (SAT) and for the dual-center 0.016 ± 0.016 (SM), 0.007 ± 0.012 (VAT), and 0.010 ± 0.015 (SAT).

### End-to-end evaluation

**Fig. 5:**
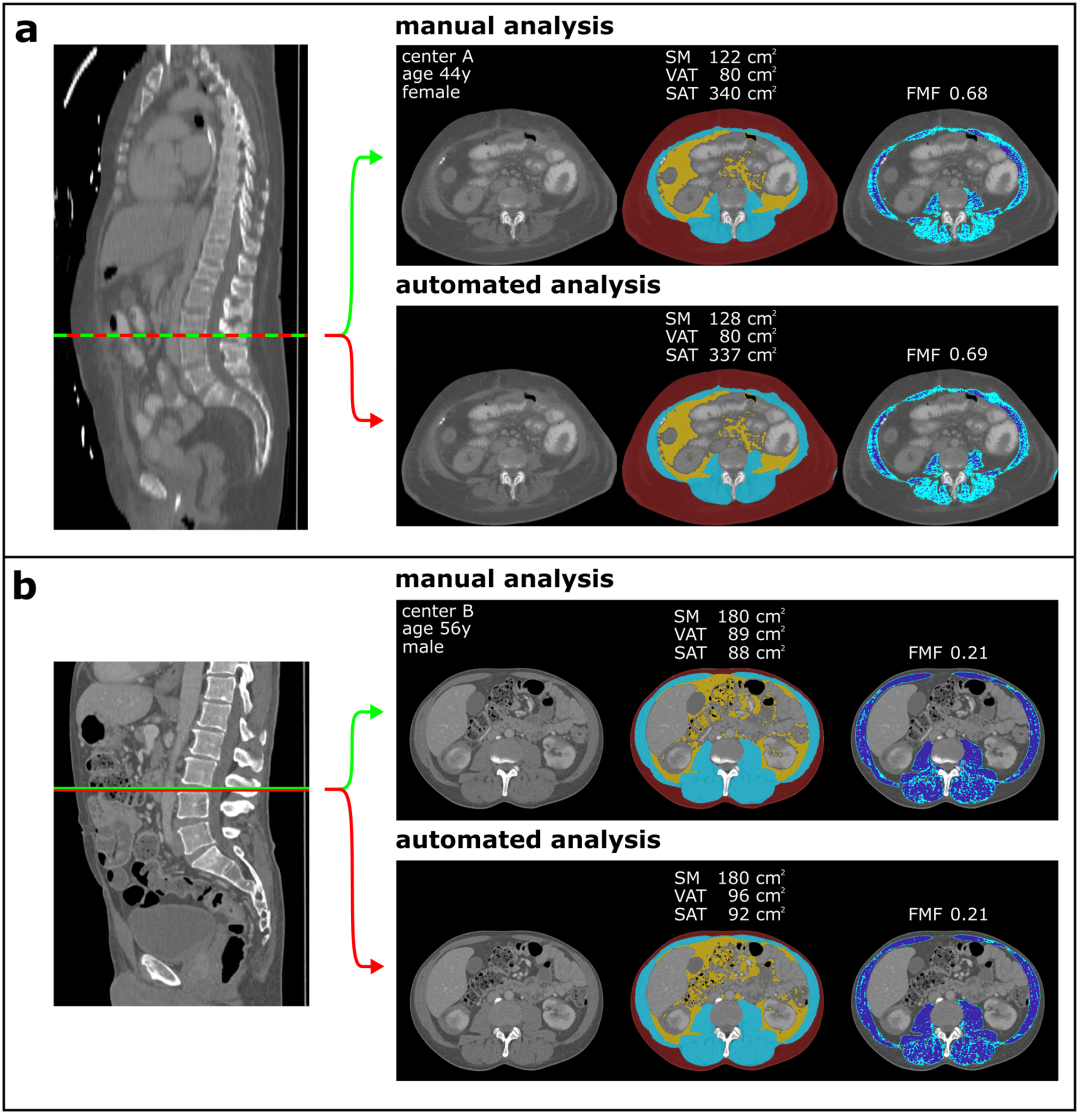
Compartmental areas of visceral adipose tissue, subcutaneous adipose tissue (VAT, SAT), skeletal muscle (SM), and fatty muscle fraction (FMF) derived for patients from center A (**a**) and center B (**b**). Manual analysis is marked in green, while results from the proposed pipeline are marked with a red line

Figure 5 shows examples of the end-to-end analyses. Application of the logistic regression model to the dual-center test data, all of which contained the L3/L4 lumbar level, resulted in 14 of 364 3D CT scans with a warning that the scan may not contain the L3/L4 level. In three of these cases, the patients had implants at the L3/L4 level. For the remaining 11 cases, the difference between predicted L3/L4 level and ground truth was Δ*z* = 6.38 ± 10.77 mm. Except for the three patients with implants, none of the patients were excluded from further analyses. Subsequently, the linear regression model for integrated quality control of the tissue segmentation was applied. With a restrictive setting, 36 of 361 cases were flagged as possibly having limited segmentation quality with predicted Dice scores of the muscle class ranging from 0.861 to 0.924. In 5 of these 36 cases, the patients had implants at the L3/L4 level, and 4 patients had a pronounced hernia. In the remaining cases, there were various reasons for limited segmentation quality, such as parts of the arms included in the tissue segmentation or parts of the kidney classified as muscle. In total, 8 of 10 cases with metallic implants on the L3/L4 level were excluded by the two quality control steps. For the two cases not excluded by quality control, only minor hardening artifacts were observed, as shown in Supplement 4S.

Results of the entire end-to-end evaluation are summarized in Table 3. A high agreement was observed for the 325 cases of the dual-center data that passed the quality control. Body composition metrics and FMF derived from automated and manual analysis showed absolute differences in area of ΔSM = 5.0 ± 6.0 cm^2^, ΔVAT = 3.7 ± 5.8 cm^2^, and ΔSAT = 5.7 ± 10.4 cm^2^, corresponding to low relative differences of ΔSM = 3.3 ± 4.1%, ΔVAT = 3.0 ± 4.7%, and ΔSAT = 2.7 ± 4.3%. Also for FMF, low absolute deviations of ΔFMF = 0.014 ± 0.012 and relative deviations of ΔFMF = 4.3 ± 4.4% were observed.

**Table 3:** Evaluation of the end-to-end performance of the body composition analyses

Center	Quality control	Fatty muscle fraction	Muscle area (cm^2^)	Visceral fat area (cm^2^)	Subcutaneous fat area (cm^2^)
A	Passed, *n* = 82	0.009 ± 0.008 (3.1% ± 3.5%)	3.7 ± 4.1 (2.7% ± 4.4%)	3.6 ± 4.3 (2.7% ± 3.6%)	5.4 ± 5.3 (2.7% ± 3.0%)
B	Passed, *n* = 243	0.016 ± 0.013 (4.8% ± 4.6%)	5.4 ± 6.4 (3.5% ± 4.0%)	3.8 ± 6.2 (3.1% ± 5.0%)	5.8 ± 11.7 (2.8% ± 4.6%)
A	Excluded, *n* = 1	**0.046 (9.3%)**	**16.0 (16.6%)**	2.0 (2.3%)	14.9 (10.8%)
B	Excluded, *n* = 35	**0.033 ± 0.036 (6.1% ± 6.6%)**	**18.6 ± 21.6 (14.1% ± 15.6%)**	7.2 ± 10.4 (7.0% ± 8.6%)	18.4 ± 29.5 (7.8% ± 9.5%)

Absolute and relative differences (in parentheses) between the values obtained with the proposed pipeline and the manually determined values are listed separately for center A and center B and for all 3D CT scans that were included and excluded by restrictive setting of the tissue segmentation quality control. The excluded cases show markedly lower agreement of muscle area, while FMF agreement is still reasonably good (marked in bold)

## Discussion

This paper presents a method that allows the application of body composition analysis without human interaction, thus permitting opportunistic determination of body compartment areas and FMF as a marker for sarcopenia in routine clinical practice. For both CNNs applied in the pipeline, the trained networks are available on reasonable request (https://qilab.de).

In recent years, a variety of deep learning methods have been presented that address the topic of automated body composition analysis. Most of these studies focus on the segmentation of the tissue compartments in a single slice at a certain lumbar level, as it has been demonstrated that 2D and 3D measurements for quantification of VAT, SAT, and SM show a high correlation [9–14]. Although very recent works have also addressed automation of slice extraction, routine clinical application additionally requires the integration of quality control methods for both slice extraction and tissue segmentation [15, 16]. For this purpose, two classic machine learning models have been developed in this study. The developed pipeline therefore provides full automation of body composition analysis in abdominal CT, including deep-learning-based slice extraction and tissue segmentation and integrated application of quality control models.

Compared to previous research in the field of automated body composition analyses, we observed similar or superior performance values for slice extraction task and tissue segmentation in our study [12–17]. In previous work, the slice extraction task was formulated either as a regression problem, a classification task, or, similar to our approach, a segmentation problem [15–17]. While the methods proposed so far for slice extraction are based on 2D images or require the generation of a maximum intensity projection in a pre-processing step, the use of the nnU-Net framework allows the direct input of 3D CT datasets of different sizes. For tissue segmentation, different variants of a 2D U-Net architecture have been used [12, 15–17]. The CDFNet architecture applied in the current study is an extension of a Dense-UNet architecture with max-out activation units, which has recently also been successfully used for body composition analyses in magnetic resonance imaging [20]. A detailed comparison to previous work can be found in Supplement S6.

For the development of the tissue segmentation CNN, patient collectives were included that also represent tissue alterations, as ascites and anasarca, which are challenging for body composition analysis [14]. In addition, segmentation results from other studies show the disadvantages of using only threshold-based pre-processing steps to define segmentation ground truth, resulting in misclassification of intermuscular fat to one of the abdominal adipose tissue classes (VAT, SAT) [15]. To overcome this limitation, intermuscular fat was manually assigned to the muscle class in this study, allowing additional analyses of muscle [13].

Several aspects of body composition, such as skeletal muscle fat infiltration as an indicator of skeletal muscle quality were shown to provide prognostic information in patients with cardiovascular and oncologic diseases [1–3]. Thereby, FMF was recently proposed as an easy-accessible body composition metric which may be considered particularly promising as it additionally integrates information on skeletal muscle quality [1, 5]. Previous studies have demonstrated its prognostic value both as an indicator of frailty in patients with planned endovascular aortic valve replacement as well as an powerful predictor of outcome in critically ill patients receiving extracorporeal membrane oxygenation therapy [1, 6].

A recent work on 3D tissue segmentation points out that for a truly automated application of body compartment analysis, the development of quality assurance procedures is warranted to identify patients with metal artifacts [24]. The dual-center end-to-end analysis presented in the current work demonstrates that the proposed quality control ensures a high agreement between manual and automated analyses by identifying cases that are unsuitable for body composition analyses not only due to hardening artifacts but also due to other reasons limiting the segmentation quality. Interestingly, end-to-end performance analysis of cases flagged by quality control as having limited segmentation quality shows that FMF is quite robust to segmentation errors.

As a limitation of this study, only the areas of VAT, SAT, and SM are determined in a single slice instead of determining the respective tissue volumes in the entire abdomen. However, we are not aware of studies demonstrating that a 3D approach has significant advantages over the established 2D measurement for assessment of sarcopenia. Also, reference values for body compartments have so far only been determined in large studies for 2D measurements [15].

## Conclusion

This study presents an end-to-end automated deep-learning pipeline for large-scale opportunistic assessment of body composition metrics and sarcopenia biomarker in clinical routine.

## Supplementary Information

Below is the link to the electronic supplementary material.

Supplementary file1 (DOCX 1273 KB)

## Abbreviations

CDFNet: Competitive dense fully connected network
CNN: Convolutional neural network
FMF: Fatty muscle fraction
SAT: Subcutaneous adipose tissue
SM: Skeletal muscle
VAT: Visceral adipose tissue
