# Supplementary material for: End-to-end automated body composition analyses with integrated quality control for opportunistic assessment of sarcopenia in CT
Source: Eur Radiol. 2021 Sep 30;32(5):3142–51. doi: 10.1007/s00330-021-08313-x (PMC9038788; doi:10.1007/s00330-021-08313-x)
Supplement: Supplementary file 1 — Supplementary file1 (DOCX 1273 KB) [file 330_2021_8313_MOESM1_ESM.docx]

**SUPPLEMENT**

**S1 Slice Extraction: Image pre-processing, augmentation and experimental design**

Prior to slice extraction, the 3D CT-scans were transformed to uniform resolution of 2 x 2 x 5mm by applying cubic interpolation. Voxel values were normalized by clipping the Hounsfield units to the 0.5 - 99.5 percentile of all foreground voxels in the entire dataset and were then transformed to a mean of zero and a standard deviation of one by the nnU-Net framework [1].

The nnU-Net framework uses extensive image augmentation while assembling the batches during training to improve the robustness of the segmentation method against overfitting. Thus, the following transformations were applied to the 3D image data: Rotation, scaling, Gaussian noise and blur, brightness, contrast, simulation of low resolution, mirroring and gamma augmentations. Detailed information on pre-processing and data augmentation of the nnU-Net framework can be found in the original work and its appendix [1]. Additionally to the data augmentation of the nnU-Net framework, all thorax/abdomen scans of the training set were cropped to abdomen and thorax/upper-abdomen scans and added to the training set. This augmentation resulted in an additional 151 thorax/upper-abdomen and 118 abdomen scans for training.

As proposed by Yang et al, the ground truth for training of the nnU-Net was created by applying a Gaussian distribution to the coordinate of the landmark [2]. Thus, a label map of the same matrix size as the image is generated by the following formula,

$$I_{gt}= \frac{1}{\sigma_{xy}\sqrt{2\pi}} {\cdot e}^{-\frac{\left( x_{1}-\mu_{1} \right)^{2}+\left( x_{2}-\mu_{2} \right)^{2}}{2\sigma_{xy}^{2}}}\cdot\frac{1}{\sigma_{z}\sqrt{2\pi}}\cdot e^{- \frac{\left( x_{3}-\mu_{3} \right)^{2}}{2\sigma_{z}^{2}}}$$

where σ_z_=1.6, σ_xy_= 8 and μ corresponds to the L3/L4 disc coordinate. The resulting probability map was then binarized by a threshold value chosen as five percent of the maximum value in the probability map. In a post-processing step, the center of mass of the nnU-Net segmentation is calculated to determine the L3/L4 level coordinate.

The used nnU-Net framework was obtained from <https://github.com/MIC-DKFZ/nnUNet>. The “3D fullres” configuration was chosen. In a first step, nnU-Net generates a fingerprint of the entire training dataset, based on which the network architecture is automatically specified [1]. This resulted in a batch size of 2 and a patch size of 160x160x80 forming the input for an architecture consisting of 5 encoder and decoder blocks with one bottleneck block. Each block consists of two convolutions with 3x3x3 kernels. Each convolution is followed by an instance normalization and a Leaky Rectified Linear Unit activation function. For downsampling a stride of 2 is applied. The only exception is the first convolutional block applied to the input patch: Due to the strong anisotropic initial spacing of 2x2x5 mm, the kernel size in z-direction is 1 for both convolutions with a stride of 1 in each dimension. A stride of 1 in z-direction is also used in the first convolution of the second block. The number of kernels are doubled in each downsampling step starting from 32 until a maximum of 320. Upsampling is performed with transposed convolutions and the encoder and decoder blocks were concatenated with skip connections.

The training was performed with mixed precision on an Nvidia Titan XP Graphics Processing Unit (GPU) with 12 gigabyte video memory. Stochastic gradient descent was used as optimization algorithm, with a nesterov momentum of μ = 0.99. A combined loss of cross-entropy and Dice loss was used for optimization. The loss is calculated as the weighted sum of losses derived at the different deep-supervision outputs of the model and thus at different resolutions. During the training of 1000 epochs, the learning rate was decayed according to the poly learning rate scheme starting from a maximum value of 0.01.

**S2 Slice Extraction: Additional Information on quality control**

To build the logistic regression model on a balanced distribution of images with and without L3/L4 lumbar level, cropped versions of all training and test 3D CT scans were generated. Cropping was performed randomly in a range up to 20cm above or below the L3/L4 level.

Both, the logistic regression model for quality control of slice extraction and the linear regression model for quality control of tissue segmentation were developed in Python using scikit-learn [3]. As with the linear regression model of tissue segmentation quality control, we used the mean entropies of the probability maps as a feature to determine the presence of the L3/L4 level based on the nnU-Net predictions used for slice extraction. In addition, we investigated the use of the predicted volume of the nnU-Net segmentation as another feature. Both logistic regression models were trained based on the prediction of all validation cases of the nnU-Net. Both features showed high performance, with an accuracy of 97% for the mean entropy and 99% for the predicted volume. The sensitivity for both features was 99%, but the specificity for the mean entropy (95%) was lower than that of the predicted volume (99%). Therefore, due to its slightly better performance, the volume of the prediction was chosen as final feature, also leading to a high performance for all test sets (c.f. Table S2).

| **hold-out test** | | | **center A** | | | **center B** | | |
| --- | --- | --- | --- | --- | --- | --- | --- | --- |
| **ACC** | **SE** | **SP** | **ACC** | **SE** | **SP** | **ACC** | **SE** | **SP** |
| 1 | 1 | 1 | 0.99 | 0.99 | 0.99 | 0.98 | 0.96 | 0.996 |

**Table S2:** Accuracy (ACC), sensitivity (SE) and specificity (SP) of the logistic regression model, classifying the presence of the L3/L4 lumbar level in the 3D CT-scan based on the total predicted volume of the segmented foreground class of the nnU-Net model.

**S3 Tissue segmentation: Image pre-processing, augmentation, experimental design and post-processing**

The extracted single slice CT images were transformed to a matrix size of 512 x 512 by applying cubic interpolation. Subsequently, image values were clipped to [-400,600] Hounsfield-Units and transformed to range zero to one [4]. Morphological opening with a kernel size of 5 was used to remove the table. Afterwards, only the largest component was extracted.

All images were augmented by affine transformations to improve the robustness of the segmentation method against overfitting. With a probability of 75%, the images were rotated by -10° to 10° around the z-axis and were scaled by 0% to 5% while assembling the batches during training.

The Competitive dense fully convolutional network (CDFNet) employed for tissue segmentation that was obtained from Github (<https://github.com/reuter-lab/FatSegNet_pytorch>) is built on four competitive encoder and four competitive decoder, and one competitive bottleneck block. Each competitive block includes two convolutions with kernel size 5x5, one convolution with kernel size 1x1, two maxout-units, batch normalizations and Parametric rectified linear unit activation (PReLU). The architecture also includes a competitive unpool block for the global skip connections between the competitive encoder and decoder blocks at each level. In the unpool block the output of the lower level decoder is upsampled using a transpose convolution and then concatenated to the output of the encoder via the skip connection. A PReLU activation, a convolution with kernel size 5x5, batch normalization and a maxout unit is applied to the concatenated feature map of the skip connection prior to the input of the competitive decoder. For all convolutional layers the number of kernels is 64. More details can be found in the original work [5, 6].

All experiments for tissue segmentation were performed in pytorch and the application programming interface fastai [7, 8]. The training was performed on an Nvidia Titan RTX Graphics Processing Unit (GPU) with 24 gigabyte video memory, using mixed precision training.

The Adam optimization algorithm, a cross-entropy loss function, a weight decay of 10^-6^, a batch size of 10 and early stopping was used for training. The learning rate and momentum followed a cyclical change during 80 epochs of training, according to the one cycle learning rate policy [9]. In detail, the learning rate was increased to a maximum value of 0.001 until 30% of the epochs were reached, starting from the maximum value divided by 25. Then the learning rate was dropped towards zero by cosine annealing until the last epoch. In contrast, the momentum was dropped from 0.95 to 0.85 and then raised back to 0.95.

As measure of muscle quality the muscular fat infiltration was quantified by calculating the fatty-muscle-fraction (FMF). Therefore, areas of fatty and lean muscle were identified using thresholds of ‑30 to 29 and 30 to 100 Hounsfield units. FMF was calculated as the fraction of fatty muscle referred to total lean and fatty muscle area [10].

**S4 Detection and Exclusion of patients with metallic implants**

Of the 364 cases of the dual-center test set, 39 cases were excluded by the quality control methods for slice extraction and tissue segmentation that included 8 out of 10 cases with metallic implants. The quality control methods did not warn in two cases with metallic implants at L3/L4 level. As shown in Figure S4, in the remaining two cases only minor artifacts are present compared to cases excluded by quality control. The predicted Dice-score for the two cases were 0.936 (difference to ground truth 0.018) and 0.938 (difference to ground truth 0.010).

If desired, all patients with metallic implants in the spine area could be easily excluded with further image processing. To demonstrate this, we developed and applied a simple image processing pipeline: first a body mask is created by applying a threshold of -400 Hounsfield Units (HU). The body mask is then cropped to the middle third in right/left direction and to the posterior third in anterior/posterior direction. Subsequently, a median filter with a kernel size 5x5 is applied. Finally, it is checked whether the maximum HU value is above 3000 HU. When applying this method to the dual-center test data, all 10 cases with implants were detected without any false positive detection.

Such a method could be used to extend quality control by explicit metal implant detection. However, due to the small number of cases with metallic implants at L3/L4 vertebral level in our study, the described approach should only be seen as an example, since its generalizability would have to be further investigated.


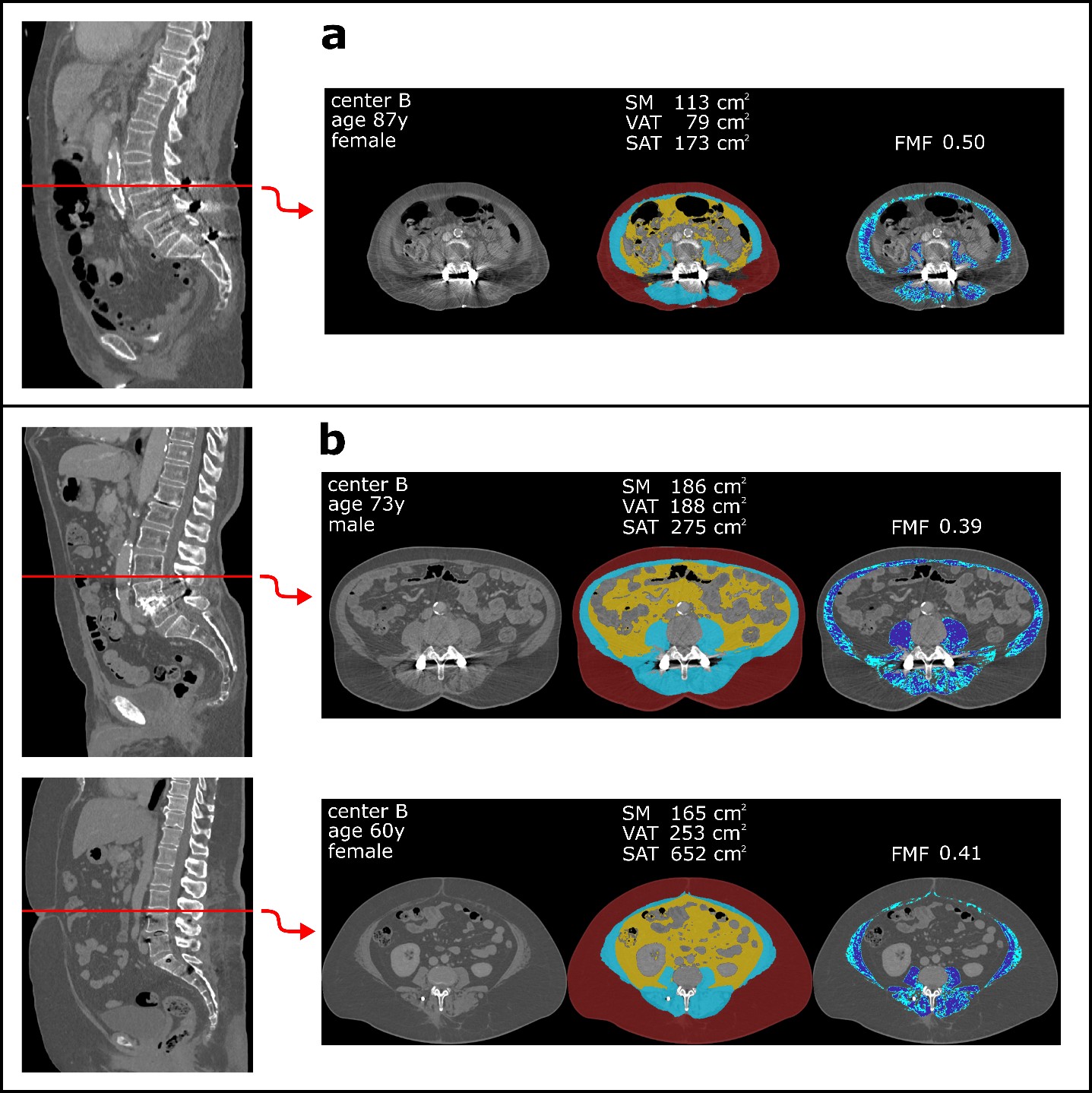


**Figure S4:** Illustration of the 2 of 10 cases with metallic implant which were not excluded by the quality control of the tissue segmentation method. Compared to a case that was excluded by the quality control (a), artifact level was markedly lower in the other two cases (b).

**S5 Additional Information on the dual-center test data**

For center A, 83 consecutive patients referred for diagnostic CT including imaging of the upper abdomen from a one-week period from four CT scanners of the radiology and neuro-radiology department were used.

300 consecutive patients from three scanners (100 patients per scanner) referred for CT including imaging of the upper abdomen were retrieved from center B. Of these 300 examinations, 19 were excluded from the analysis because they were entirely interventional CT scans. In the case of a multiphase CT-examination, one phase that included the entire abdomen was selected randomly.

Table S5.1 lists the number of scans included and the respective percentage of data from the different CT scanners. Typical imaging parameters are listed in Table S5.2.

| **Vendor** | Philips | | | Siemens | | |
| --- | --- | --- | --- | --- | --- | --- |
| **CT model** | iCT 256 | iCT SP | IQon – Spectral CT | SOMATOM Definition AS+ | SOMATOM Definition Flash | SOMATOM Force |
| **Center A**  **(N=83)** | 30% | 34% | 7% | - | - | 29% |
| **Center B**  **(N=281)** | - | - | - | 35% | 31% | 34% |

Table S5.1: Number of cases per center and percentage of data from different CT-scanners.

|  | **Center A (N=83)** | | | **Center B (N=281)** | | |
| --- | --- | --- | --- | --- | --- | --- |
|  | mean | median | range | mean | median | range |
| tube voltage [kV] | 113 | 120 | [90; 120] | 100 | 100 | [70; 150] |
| slice thickness [mm] | 4.9 | 5.0 | [4.0; 5.0] | 1.3 | 1.3 | [0.7; 1.5] |
| pixel spacing [mm] | 0.82 | 0.79 | [0.64; 0.98] | 0.82 | 0.82 | [0.58; 0.98] |
| matrix size | 512 | 512 | [512; 512] | 512 | 512 | [512; 512] |
| number of slices | 124 | 129 | [65; 195] | 385 | 357 | [246; 1936] |

**Table S5.2:** Image parameters of the dual-center test data.

**S6 Comparison to previous work**

**Comparison of different approaches for slice extraction:**

In the current work, the slice extraction task was formulated as a 3D segmentation problem, which facilitated the application of the nnU-Net framework [1]. This framework has achieved high performance values on various medical segmentation tasks and also has the advantage of allowing automatic architecture adaptation to different input sizes.

In some previous work on slice extraction the task has been formulated as a binary classification or a regression [11, 12]. Dabiri et al. proposed an ImageNet pre-trained VGG-11 model that classifies each slice as "L3" or "non-L3" based on 2D axial slices [11]. Magudia et al. used a DenseNet architecture to predict the distance between an axial slice and the L3 lumbar plane [12].

Similar to our approach, Castiglione et al. used a U-Net-based architecture to extract the L3 layer from a CT dataset. However, the pipeline requires the conversion of the 3D CT dataset into a 2D maximum intensity projection (MIP) in a pre-processing step. The model of Castiglione et al. was pre-trained on three different publicly available CT datasets, most of which were from adult patients, while the final model was trained specifically for a paediatric study population of children aged 0-18 years [13].

Table S6.1 summarises the results of previously published slice extraction methods and the results of the current study.

| **Work/Method** | **Patient cohort** | **Result** | |
| --- | --- | --- | --- |
| Dabiri et al.  2D VGG-11 | *Dataset:*   - Dataset-1: 2D axial slices at L3 level from patients with head, neck and lung cancer - Dataset-2: 3D CT scans of patients with invasive colorectal cancer after surgical resection - Dataset-3: 2D CT slices at L3 level from female patients with invasive breast cancer   *( Training:* >6600 slices for each class “*L3”* and “*non-L3*” (60% from dataset-2; 100% from dataset-1 and dataset-3, Test*:* 1748 3D CT scans (40% from dataset-2) ) | n  Mean slice error  Mean error [mm]  Median error [mm]  Maximum error [mm] | 1748  $0.87\pm2.54$  -  -  - |
| Magudia et al.  2D DenseNet | *Dataset:* 604 patients with pancreatic adenocarcinoma (421 training, 94 validation, 89 test)  *Additional Test:* external dataset with 534 abdominal CT examinations from patients with lymphoma | n  Mean slice error  Mean error [mm]  Median error [mm]  Maximum error [mm] | 534  -  $11$  $5$  $40$ |
| Castiglione et al.  2D U-Net | *Dataset:* 370 paediatric patients, age 0 to 18 years (296 training, 74 test) | n  Mean slice error  Mean error [mm]  Median error [mm]  Maximum error [mm] | 74  -  $3.23\pm2.61$  -  - |
| Proposed approach  3D nnU-Net | *Dataset:* 240 CT-scans including upper abdomen (192 training, 48 test)  *Additional (add.) test:* consecutive patients from two centers | **Test**  n  Mean slice error  Mean error [mm]  Median error [mm]  Maximum error [mm] | 48  $0.45\pm1.42$  $2.27\pm7.08$  $0.04$  $35.27$ |
|  |  | **Add. Test**  n  Mean slice error  Mean error [mm]  Median error [mm]  Maximum error [mm] | 364  $0.49\pm1.24$  $2.46\pm6.20$  $0.10$  $40.00$ |

**Table S6.1:** Summary of the results of various slice extraction methods.

Compared to other work on slice extraction in CT, the method presented in this work showed the lowest mean error on the test set. Note also that the work of Castiglione et al. focuses on paediatric patients, where the absolute distances between different vertebrae are smaller compared to adults [13].

**Comparison of different approaches for tissue segmentation:**

In our work, we used a CDFNet for tissue segmentation of skeletal muscle (SM), subcutaneous adipose tissue (SAT) and visceral adipose tissue (VAT) in a 2D axial slice at the L3/L4 lumbar level. The CDFNet uses a novel feature combination approach that has been shown to have performance and generalisability advantages over established approaches (standard U-Net, DenseNet). Promising results have recently been shown for fat tissue segmentation on MRI using a CDFNet [5, 6].

Previous work employs different variants of the U-Net architecture for the tissue segmentation task. Dabiri et al. used a U-Net-like architecture with four convolutional layers in the encoder and four in the decoder, which is extended by three up-sampling layers in parallel with the decoder and three dilated convolutional layers at the end of the architecture [11].

Magudia et al. used a classical U-Net architecture consisting of five convolutional layers in the encoder, five in the decoder and a bottle-neck block [12].

Similar to these approaches, Castiglione et al. used a 2D U-Net architecture consisting of four convolutional layers in the encoder, four in the decoder and a bottle-neck block. They compared four different models, one with binary cross-entropy loss and the other with a weighted Dice similarity coefficient loss function (DSC), each trained once with and once without data augmentation. The model with the highest performance was trained without data augmentation and with a weighted DSC loss [13].

Like Magudia et al., Weston et al. used a 2D U-Net based architecture with five upsampling, five downsampling blocks and a bottle-neck block with two convolutions each. In contrast to the standard U-Net architecture, the hyperbolic-tangent activation function was used instead of rectified linear unit [4].

In addition to work that also uses fully connected 2D U-Net like architectures, we compared our method to a previously published work where an adaptation of the DeepMedic architecture, called Deep19, was used [14].

The open source package DeepMedic uses a hybrid scheme of patchwise and dense training on entire images [15]. Unlike the standard U-Net scheme, DeepMedic does not use downsampling layers within the architecture. Instead, the architecture consists of two or more parallel paths that receive the input image in different resolutions, which are eventually fused in fully connected layers. The Deep19 model consists of two paths with 19 convolutional layers each. One path receives a patch with original and the other a patch with image resolution lowered by factor 6. The 2D CDFNet presented in this work was trained on the same data as the Deep19 model. The results are summarised in Table S6.2.

| **Work/Method** | **Patient cohort** | **Result** | | |
| --- | --- | --- | --- | --- |
| Dabiri et al.  2D U-Net | *Dataset*:   - Dataset-2: 3D CT scans of patients with stage 3 invasive colorectal cancer after surgical resection - Dataset-3: 2D CT slices at L3 level from female patients with stage 2 or 3 invasive breast cancer   *Training dataset-2:* 3774 L3 slices from dataset-2  *Training dataset-3:* 1801 L3 slices from dataset-3  *Test dataset-2:* 1327 L3 slices from dataset-2  *Test dataset-3:* 1202 L3 slices from dataset-3 |  | **Trained on dataset-2** | **Trained on dataset-3** |
|  |  | **Test dataset-2**  n  Dice SM  Dice VAT  Dice SAT | 1327  $0.99\pm0.01$  $0.99\pm0.04$  $0.99\pm0.04$ | 1327  $0.97\pm0.02$  $0.98\pm0.05$  $0.98\pm0.04$ |
|  |  | **Test dataset-3**  n  Dice SM  Dice VAT  Dice SAT | 1202  $0.97\pm0.03$  $0.97\pm0.04$  $0.99\pm0.01$ | 1202  $0.98\pm0.02$  $0.98\pm0.04$  $0.99\pm0.01$ |
| Magudia et al.  2D U-Net | *Dataset:* 604 patients with pancreatic adenocarcinoma (421 training, 94 validation, 89 test) | n  Dice SM  Dice VAT  Dice SAT | 89  $0.97\pm0.03$  $0.95\pm0.10$  $0.98\pm0.02$ | |
| Castiglione et al.  2D U-Net | *Dataset:* 370 pediatric patients, age group from 0 to 18 years (296 training, 74 test) | n  Dice SM  Dice VAT  Dice SAT | 74  $0.93\pm0.03$  -  - | |
| Weston et al.  2D U-Net | *Dataset:* 2700 CT images from 1429 patients with different cancer types (2187 training, 243 validation, 270 test)  *Additional (add.) test:* 2369 CT images from 1083 patients with HCC | **Test**  n  Dice SM  Dice VAT  Dice SAT | 270  $0.96\pm0.02$  $0.94\pm0.12$  $0.98\pm0.03$ | |
|  |  | **Add. Test**  n  Dice SM  Dice VAT  Dice SAT | 2369  $0.92\pm0.04$  $0.98\pm0.02$  $0.94\pm0.05$ | |
| BLINDED  Deep19 | *Dataset:* CT slices at L3/L4 level of 1143 consecutive patients with preinterventional CT for TAVI or diagnostic CT for liver cirrhosis with portosystemic shunting (PTSS) (801 training, 171 validation, 171 test)  *Additional test:*  *Test 1:* 100 randomly selected patients with routine diagnostic abdominal CT  *Test 2:* 50 consecutive patients with native CT examinations | **Test**  n  Dice SM  Dice VAT  Dice SAT | 171  $0.95\pm0.02$  $0.96\pm0.04$  $0.98\pm0.01$ | |
|  |  | **Add. Test 1**  n  Dice SM  Dice VAT  Dice SAT | 100  $0.95\pm0.04$  $0.95\pm0.05$  $0.97\pm0.04$ | |
|  |  | **Add. Test 2**  n  Dice SM  Dice VAT  Dice SAT | 50  $0.97\pm0.02$  $0.97\pm0.03$  $0.99\pm0.01$ | |
| Proposed approach  2D CDFNet | *Dataset:* CT slices at L3/L4 level of 1143 consecutive patients with preinterventional CT for TAVI or diagnostic CT for liver cirrhosis with portosystemic shunting (PTSS)  (972 training (5-fold cross-validation), 171 test)  *Additional test:*  364 consecutive patients from two centers | **Test**  n  Dice SM  Dice VAT  Dice SAT | 171  $0.96\pm0.02$  $0.98\pm0.02$  $0.98\pm0.01$ | |
|  |  | **Add. Test**  n  Dice SM  Dice VAT  Dice SAT | 364  $0.95\pm0.04$  $0.98\pm0.02$  $0.97\pm0.04$ | |

**Table S6.2:** Summary of the results of various tissue segmentation methods.

From Table S6.2, it can be seen that our work shows high performance compared to other research in this area. The CDFNet we used for tissue segmentations shows slightly better performance for the segmentation of SM and VAT and equal performance for segmentation of SAT compared to our previous work, which was tested on the same hold-out test set [14]. Also, compared to Weston et al., Magudia et al. and Castiglione et al., a higher or equal Dice coefficient was obtained for each tissue class, except for the segmentation of SM by Magudia et al., which scored higher on the hold-out test set [4, 12, 13]. In the work of Dabiri et al. segmentation performance was highest for most tissue classes, which may be at least partly addressed to the dataset rather than the method itself [11]. Our dataset included data from patients with ascites, anasarca or both ascites and anasarca. These were intentionally not excluded in our work, as these conditions are known to complicate body composition analysis and are frequently observed in clinical routine.

**References**

1. Isensee F, Jaeger PF, Kohl SAA, Petersen J, Maier-Hein KH (2021) nnU-Net: a self-configuring method for deep learning-based biomedical image segmentation. Nat Methods 18:203–211.

2. Yang D, Xiong T, Xu D, et al (2017) Deep Image-to-Image Recurrent Network with Shape Basis Learning for Automatic Vertebra Labeling in Large-Scale 3D CT Volumes. Proceedings of MICCAI 2017, pp 498–506.

3. Pedregosa F, Varoquaux G, Gramfort A, et al (2011) Scikit-learn: Machine Learning in Python. J Mach Learn Res 12:2825–2830

4. Weston AD, Korfiatis P, Kline TL, et al (2018) Automated Abdominal Segmentation of CT Scans for Body Composition Analysis Using Deep Learning. Radiology 290:669–679.

5. Estrada S, Lu R, Conjeti S, et al (2020) FatSegNet: A fully automated deep learning pipeline for adipose tissue segmentation on abdominal dixon MRI. Magn Reson Med 83:1471–1483.

6. Estrada S, Conjeti S, Ahmad M, Navab N, Reuter M (2018) Competition vs. Concatenation in Skip Connections of Fully Convolutional Networks. Proceedings of International Workshop on Machine Learning in Medical Imaging, pp 214–222.

7. Paszke A, Gross S, Massa F, et al (2019) PyTorch: An Imperative Style, High-Performance Deep Learning Library. arXiv:191201703

8. Howard J, Gugger S (2020) Fastai: A Layered API for Deep Learning. Information 11:108.

9. Smith LN (2018) A disciplined approach to neural network hyper-parameters: Part 1 -- learning rate, batch size, momentum, and weight decay. arXiv:180309820

10. Luetkens JA., Faron A, Geissler HL., et al (2020) Opportunistic Computed Tomography Imaging for the Assessment of Fatty Muscle Fraction Predicts Outcome in Patients Undergoing Transcatheter Aortic Valve Replacement. Circulation 141:234–236.

11. Dabiri S, Popuri K, Ma C, et al (2020) Deep learning method for localization and segmentation of abdominal CT. Comput Med Imaging Graph 85:101776.

12. Magudia K, Bridge CP, Bay CP, et al (2020) Population-Scale CT-based Body Composition Analysis of a Large Outpatient Population Using Deep Learning to Derive Age-, Sex-, and Race-specific Reference Curves. Radiology 298:319–329.

13. Castiglione J, Somasundaram E, Gilligan LA, Trout AT, Brady S (2021) Automated Segmentation of Abdominal Skeletal Muscle on Pediatric CT Scans Using Deep Learning. Radiol Artif Intell 3:e200130.

14. BLINDED

15. Kamnitsas K, Ledig C, Newcombe VFJ, et al (2017) Efficient multi-scale 3D CNN with fully connected CRF for accurate brain lesion segmentation. Med Image Anal 36:61–78.
